# Improvement of Physicomechanical Properties of Pineapple Leaf Fiber Reinforced Composite 

**DOI:** 10.1155/2018/7384360

**Published:** 2018-06-12

**Authors:** K. Z. M. Abdul Motaleb, Md Shariful Islam, Mohammad B. Hoque

**Affiliations:** ^1^Department of Textile Engineering, BGMEA University of Fashion and Technology, Dhaka, Bangladesh; ^2^Department of Textile Engineering, World University of Bangladesh, Dhaka, Bangladesh

## Abstract

Pineapple leaf fiber (PALF) reinforced polypropylene (PP) composites were prepared by compression molding. The fiber content varied from 25% to 45% by weight. Water uptake percentages of the composites containing various wt% of fiber were measured. All the composites demonstrated lower water uptake percentages and maximum of 1.93% for 45 wt% PALF/PP composite treated with 7(w/v)% NaOH. Tensile Strength (TS), Tensile Modulus (TM), Elongation at Break (Eb %), Bending Strength (BS), Bending Modulus (BM), and Impact Strength (IS) were evaluated for various fiber content. The 45 wt% PALF/PP composite exhibited an increase of 210% TS, 412% TM, 155% BS, 265% BM, and 140% IS compared to PP matrix. Moreover, with the increasing of fiber content, all the mechanical properties increase significantly; for example, 45 wt% fiber loading exhibited the best mechanical property. Fibers were also treated with different concentration of NaOH and the effects of alkali concentrations were observed. The composite treated with 7 (w/v)% NaOH exhibited an increase of 25.35% TS, 43.45% TM, 15.78% BS, and 52% BM but 23.11% decrease of IS compared to untreated composite. Alkali treatment improved the adhesive characteristics of fiber surface by removing natural impurities, hence improving the mechanical properties. However, over 7% NaOH concentration of the tensile strength of the composite reduced slightly due to overexposure of fibers to NaOH.

## 1. Introduction

Use of natural fibers in composite fabrication drew great interest from the researchers due to its biodegradability and acceptable mechanical strength. As a matrix material, polypropylene has been extensively used with natural fiber in composite preparation [1, 2]. After banana and citrus fruit, pineapple (*Ananas comosus*) is one of the most essential tropical fruits in the world [3]. Hence leaves of pineapple can be used for producing natural fibers, which necessarily are considered as waste materials. Pineapple leaf fibers (PALF) are composed of holocellulose (70–82%), lignin (5–12%), and ash (1.1%), with tremendous mechanical properties [4]. Composites, the wonder lightweight material with high strength-to-weight ratio and stiffness properties, have come a long way in replacing conventional materials like metals, woods, etc. [5].

Researchers are having great interest in finding out new sources of raw materials that possess comparable physical and mechanical properties to synthetic fibers. Various other parameters to be considered while selecting raw materials are being cheap, being ecofriendly [6], absence of health hazards, high degree of flexibility [7], lower plant's age, easy collection, and regional availability which directly influence the suitability of natural fibers [8]. Above all the natural fibers are renewable resource, thus providing a better solution of sustainable supply; for example, it has low cost, low density, least processing expenditure, no health hazards, and better mechanical and physical properties [9].

Thermoplastic matrix materials are the most important part of a composite. Polypropylene (PP) is an amorphous thermoplastic polymer and is widely used as an engineering thermoplastic, as it possesses useful properties such as transparency, dimensional stability, flame resistance, high heat distortion temperature, and high impact strength. PP is also very suitable for filling, reinforcing, and blending. PP with fibrous natural polymers of biomass origin is one of the most promising routes to create natural-synthetic polymer composites [10].

However, the major limitation of natural fibers/polymers composites is the incompatibility between the hydrophobic thermoplastic matrices and the hydrophilic natural fibers [11]. Therefore it is highly important to modify the natural fiber surface to increase the fiber and matrix interaction. Chemical treatments, graft copolymerization, and use of coupling agents on natural fibers have already been explored by a number of researchers with a view to improve the fiber/matrix interaction [12]. However, mercerization of natural fibers by alkali is the most popular method nowadays to improve fiber/matrix interactions by reducing the hydrophilicity of the natural fiber. In this study, sodium hydroxide (NaOH) is used for the surface modification of the PALF.

PALF has been explored by a number of researchers in the fiber reinforced composites, and they have various alkali treatment with a view to improve the mechanical properties of the composite. However, the effect of the alkali concentration has not been explored. Therefore, the aim of this study is to manufacture the PALF/PP composite having better mechanical strength. The effects of fiber loading on the mechanical properties of PALF/PP composites are analyzed. Finally, the effects of the concentration of the alkali treatments on the mechanical properties of the composite are evaluated.

## 2. Materials and Methods

### 2.1. Materials

Granules of PP were purchased from the Cosmoplene Polyolefin Company Ltd. (401, Ayer Merbau Road, Singapore). Raw pineapple fibers were collected from Tangail, Bangladesh.

### 2.2. Alkali Treatment

Pineapple fibers were washed multiple times to remove the dirt and impurities attached on the fiber surface. After drying, the fibers were cut into small pieces. NaOH solutions were prepared with three different concentrations as 3, 5, and 7 (w/v) %. Then the fibers were emerged on NaOH solution and treated for 1 hour at room temperature, maintaining the fiber to solution weight ratio at 1:20 (w/v). Treatment with NaOH helps to remove the hydrogen bonding in the network structure of the fibers cellulose, thereby increasing fibers surface roughness [13]. Certain amount of wax and oils covering the external surface of the fibers cell wall, as well as lignin, will be removed in this process. Besides, this process will expose the short length crystallites and depolymerise the native cellulose structure [14].

After 1 hour samples were washed thoroughly until the neutral pH is achieved. Then the fibers were dried by oven at 80°C for 20 hours and stored in the plastic container.

### 2.3. Fabrication of Composite

PP sheet was prepared from its granules (10 g) by heat press. The press was operated at 360°F. Steel plates were pressed for 5 min under a pressure of 2 tons. The plates were then cooled for 5 min in a cold press under the same pressure at room temperature. The resulting PP sheet was cut into desired size for composite fabrication. Composites were prepared by sandwiching pineapple fibers between two sheets of PP. The fiber content in the pineapple composites were 25%, 30%, 35%, 40%, and 45% by weight. The prepared composites were then packaged in polyethylene bags prior to testing.

### 2.4. Sample Identification

Thickness of the samples was recorded by digital slide calipers. Average results of three readings from different place along the sample have been taken for measuring each thickness. Different samples are marked as mentioned in Table 1.

### 2.5. Water Uptake Test

Water uptake test was carried out according to ASTM D-570. Composite samples were immersed in a beaker containing 100 ml of deionized water at room temperature (25°C) for 1 hour. Initially, weight of the samples was determined, after certain time interval; samples were taken out of the beaker and wiped using tissue papers. Their weight was taken again. In this case, it shows no uptake after 50 minutes; that is why we carried out the test up to 1 hour [19]. Water uptake test was carried out on three samples of different weight of each composite. Water uptake percentage was determined by using the following equation:

(1) $$\begin{array}{c} \text{Water up-take }\left(\;\%\;\right)=\left(\;\frac{W_{f}-W_{i}}{W_{f}}\;\right)\times 100\% \end{array}$$

where* W*_i_ is initial weight (oven dry weight) and W_f_ is final weight (weight after immerse in water).

### 2.6. Mechanical Test

The tensile strength (TS), Young's modulus (YM), and elongation at break (EB%) of the composites were measured according to the European standard (ISO/DIS 527-1:2010) by a universal tensile testing machine (H50 KS-0404) with an initial clamp separation of 20mm and a cross-head speed of 10 mm/min speed. The samples sizes were 60mm×10mm×1.6mm. The samples were conditioned at 25°C and 50% relative humidity for two days before testing and all the tests were performed under the same conditions. The samples were conditioned at 25°C and 50% relative humidity for two days before testing and all the tests were performed under the same conditions (ISO 291:2012) for consistent result.

Equations (2), (3), and (4) were used for measuring the tensile strength, elongation at break percentage, and Young's modulus, respectively.

(2) $$\begin{array}{c} \text{Tensile strength},\;\;\sigma =\frac{Force}{Area}=\frac{F_{max}}{A} \end{array}$$

where *F*_max_ is maximum load applied to the sample and A is cross-sectional area of the sample.

Percentage of elongation at break was obtained by the following relation:

(3) $$\begin{array}{c} \text{Elongation at Break},\;EB\left(\;\%\;\right)=\left(\;\frac{{\Delta L}_{b}}{L_{0}}\;\right)\times 100 \end{array}$$

where ΔL_b_ is extension at break point and L_0_ is original length of the sample.

(4) $$\begin{array}{c} \text{Young's Modulus},\;\;Y=\frac{d\sigma }{d\varepsilon } \end{array}$$

where d*σ* is stress at yield point and d*ε* is strain at yield point.

Bending test was carried out for determining bending strength (BS) and bending modulus (BM) using the same universal tensile testing machine according to ISO standard (ISO 14125).

Bending test was carried out for determining bending strength (BS) and bending modulus (BM) using the same universal tensile testing machine.

Bending strength and modulus were calculated by (5) and (6), respectively.

(5) $$\begin{array}{c} \text{Bending Strength},\;\;\sigma =\frac{3FL}{2bd^{2}} \end{array}$$

*where F* is the load (force) at the fracture point (N),* L* is the length of the support span,* b* is width, and* d* is thickness.

(6) $$\begin{array}{c} \text{Bending Modulus},\;\;E_{bend}=\frac{L^{3}F}{4wh^{3}d} \end{array}$$

where* w* and* h* are the width and height of the sample,* L* is the distance between the two outer supports, and* d* is the deflection due to the load* F* applied at the middle of the sample.

Dynamic impact test was conducted to evaluate impact strength (IS) on unnotched mode composite specimens according to ASTM D 6110-97 standard using an Impact Tester (HT-8041B IZOD, Pendulum type, Taiwan). Mechanical property measurement for each composite was repeated for four times for accuracy, and three duplicate samples having the same dimensions were tested.

## 3. Results and Discussion

### 3.1. Water Uptake

**Figure 1:**
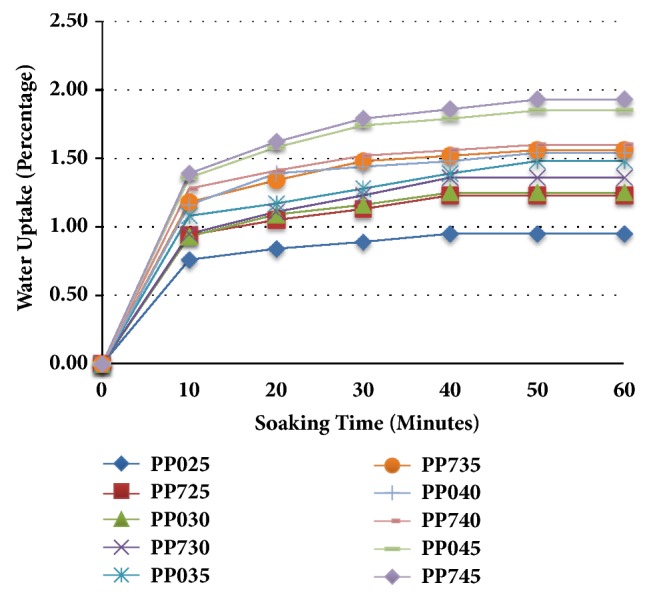
Water uptake percentage of PALF/PP composites.

Water uptake determines the water-swelling behavior of the composites. Water uptake test was carried out on three samples of each composite and their average values were calculated. The results of water uptake are shown in Figure 1. It can be observed from Figure 1 that the water absorption percentages were increased with the increasing soaking time.

The maximum water absorption was found when the soaking time was about 50 minutes for almost all the composites and, in most of the cases, water absorption was very fast (almost 50% of maximum water uptake) in the first 10 minutes of soaking time; then it became slower gradually. It is also found that alkali-treated composite takes more water than untreated composites; for example, sample PP725 took 29% more water than sample PP025 at maximum level.

Alkali treatment removes all the noncellulosic impurities, so microgaps may be increased due to the removal of interfibrillar matrix material, such as lignin and pectin. Then the small water molecules may penetrate fiber surface easily due to having more space, resulting in more water absorption by the alkali-treated composite [19].

Besides, with the increase of fiber content (wt%) the water uptake percentage was increased; for example, sample PP045 took 94.73% more water compared to sample PP025 at maximum level. The highest water uptake percentage (1.93%) was recorded for sample PP745 at 50 minutes of soaking time. With the increased fiber loading in the composite, more absorbent sites are available, and more empty spaces resulting from the removal of the lignin and pectin are present, which leads to the higher water uptake [19].

### 3.2. Effect of Fiber Loading on Mechanical Properties

**Figure 2:**
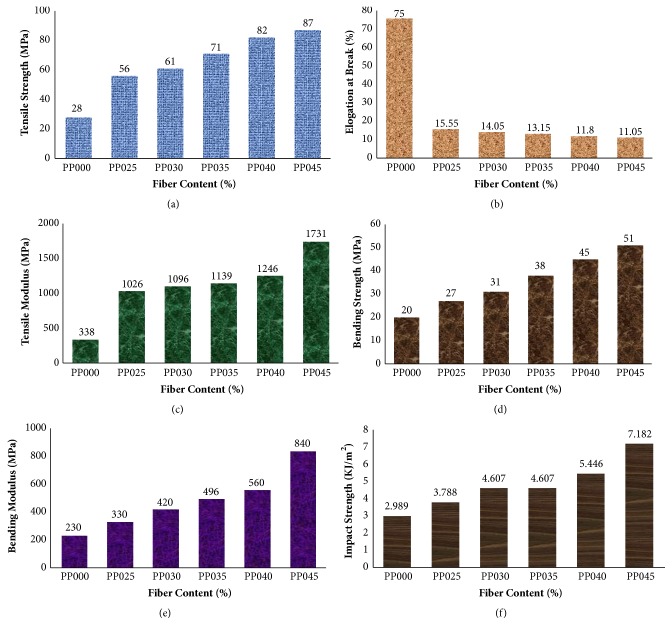
Effect of fiber content on the mechanical properties of PALF/PP composites.

Tensile strength (TS), tensile modulus (TM), elongation at break (Eb%), bending strength (BS), bending modulus (BM), and impact strength (IS) of the prepared composites were studied and the data are given in Figure 2. The TS, TM, and Eb% of PP matrix were found to be 28 MPa, 338 MPa, and 75%, respectively, for sample PP025 (25 wt% fiber). Composite with 40 wt% fibers content (sample PP040) showed an increase of 192% TS and 268% TM. In all cases of fiber addition to composite, Eb% was lowered compared to PP. This was because PALF in general have low elongation percentage compared to PP [20]. So, the elasticity of polypropylene decreases with the addition of pineapple fiber and, therefore, the elongation at break also decreases which shows that composite became brittle after the increase in volume. It was noticed that composite with 45 wt% fiber content (PP045) brought out higher BS and BM values. The results revealed that, due to 45 wt% reinforcement by PALF (PP045), an increase of 155% BS and 265% BM was observed. The IS values of PP045 were obtained 7.182 kJ/m^2^. It was noticed that 140% IS was increased for PP045 compared to PP025. From Figure 1, it is clear indication that PP000 < PP025 < PP030 < PP035 < PP040 < PP045 for all the properties except elongation at break. That means that, with the increasing of fiber content, all the mechanical properties increase. PALF composites gained huge mechanical properties over the matrix material and thus indicated good fiber matrix adhesion.

The superiority of mechanical properties of pineapple leaf fiber is related to high content of alpha-cellulose content with low microfibrillar angle (14°). As reinforcing agent PALF has both qualities, that is, high content of alpha-cellulose content with low microfibrillar angle (14°), the result of PALF based polymer composites shows excellent stiffness and strength compared to other cellulose based composite materials.

### 3.3. Effect of Alkali Concentration on Mechanical Properties

**Figure 3:**
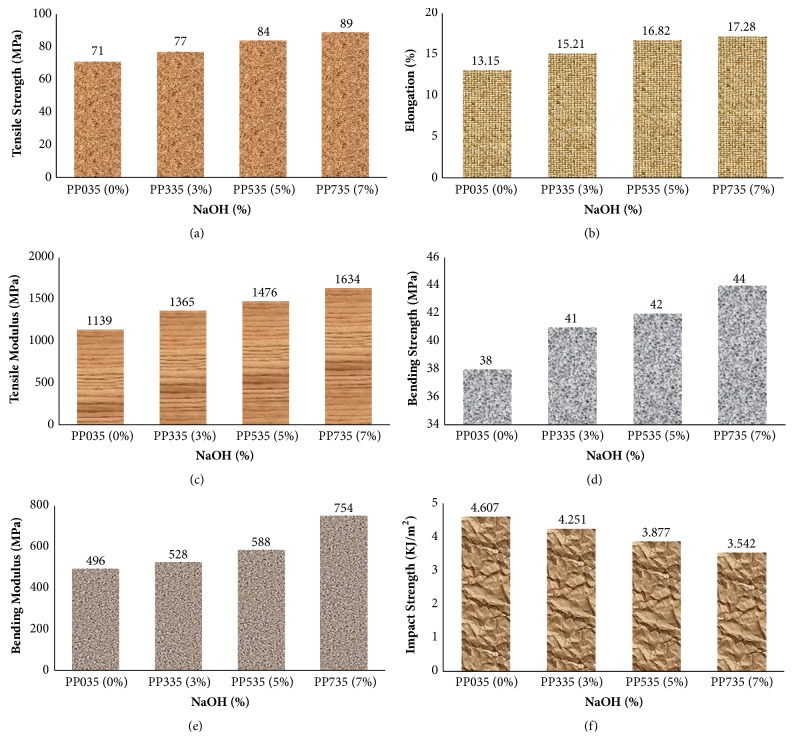
Effect of alkali treatment on the mechanical properties of PALF/PP composites.

Figure 3 depicts the TS, EB, TM, BS, BM, and IS of alkali-treated and alkali-untreated PALF/PP composites.

Before PALF was treated with 3, 5, and 7 (w/v) % of NaOH, in all the cases fiber content was 35 wt%. Then all the mechanical properties of all the samples were measured. The samples were found as PP035 < PP335 < PP535 < PP735 for all the properties. For example, TS, EB, and TM were increased 25.35%, 31.4%, and 43.45%, respectively, for sample PP735 compared to sample PP035. It is observed that the bending strength and modulus were also increased by 15.78% and 52%, respectively, but the impact strength was decreased 23.11% for sample PP735 compared to sample PP035.

From the figure it is clear that alkali treatment improves the mechanical properties of the PALF reinforced polypropylene composites. It is also evident that the percentage of NaOH is directly related to all the mechanical properties and composite, treated with 7 (w/v) % NaOH showing better mechanical properties. The increasing of tensile strength and modulus value of alkali-treated composites is due to the fact that the alkali treatment improved the adhesive characteristics of fiber surface by removing natural and artificial impurities.

Moreover, alkali treatment leads to fiber fibrillation, that is, breaking down of the fiber bundles into smaller fibers and results in the increasing of interface between matrix and fiber [21, 22]. This also increases in the number of reactive sites and changes in chemical compositions of the fibers [23]. The improvement of the mechanical properties may also be achieved by removing hemicellulose and lignin contents from the fiber [24]. However, when the composite was treated with 10% NaOH, it reduces the tensile strength of the composite to 81 MPa. The reason for this may be that the higher alkali concentration weakens the fiber by excess delignification [25].

## 4. Comparison of the Tensile Strength of PALF/PP Composite with Other Composites

It can be seen from Table 2 that PALF/PP fiber exhibits superior tensile strength than other natural fiber reinforced composites. Therefore PALF/PP fiber can be a potential natural fiber reinforced composite for various applications.

## 5. Conclusions

Pineapple leaf fiber is very common in tropical regions and it is very simple to extract fibers from its leaves. The utilization of pineapple leaf fiber in composite material is a new source of materials which can be economic, ecofriendly, and recyclable. It was found that PALF/PP composite shows better tensile strength than other natural fiber reinforced composites. Besides, better mechanical properties of composite were observed with the increase of fiber loading and alkali treatment. Moreover, water uptake percentage increased with alkali treatment. Thus, PALF may be used in fabrication of ecofriendly composite products for diversified applications and, thus, synthetic fibers can easily be replaced with PALF.

## Figures and Tables

**Table 1:** Sample Identification.

**Sl. No.**	**Samples**	**Thickness** **(mm)**	**Identification Code**			
			**0**%** NaOH**	**3**%** NaOH**	**5**%** NaOH**	**7**%** NaOH**
01	PP Matrix	0.8	PM000	-	-	-
02	25 wt% PALF/PP composite	1.5	PP025	PP325	PP525	PP725
03	30 wt% PALF/PP composite	1.66	PP030	PP330	PP530	PP730
04	35 wt% PALF/PP composite	1.73	PP035	PP335	PP535	PP735
05	40 wt% PALF/PP composite	1.85	PP040	PP340	PP540	PP740
06	45 wt% PALF/PP composite	1.97	PP045	PP345	PP545	PP745

**Table 2:** Comparison of mechanical properties.

Sl.	Name of the composite	Tensile strength (MPa)	References
1	PALF/PP	89	This study
2	Jute-Coir hybrid fibre/PP	27	[15]
3	Jute fibre/PP	55	[16]
4	Banana fibre/Epoxy resin	45.57	[17]
5	Jute-Banana hybrid fibre/Epoxy resin	18.96	[18]
6	Palm-Coir hybrid fiber/PP	30	[11]
